# Fascioliasis Control: *In Vivo* and *In Vitro* Phytotherapy of Vector Snail to Kill *Fasciola* Larva

**DOI:** 10.1155/2011/240807

**Published:** 2011-10-23

**Authors:** Kumari Sunita, D. K. Singh

**Affiliations:** Malacology Laboratory, Department of Zoology, DDU Gorakhpur University, Gorakhpur 273009, India

## Abstract

Snail is one of the important components of an aquatic ecosystem, it acts as intermediate host of *Fasciola* species. Control of snail population below a certain threshold level is one of the important methods in the campaign to reduce the incidence of fascioliasis. Life cycle of the parasite can be interrupted by killing the snail or *Fasciola* larva redia and cercaria in the snail body. *In vivo* and *in vitro* toxicity of the plant products and their active component such as citral, ferulic acid, umbelliferone, azadirachtin, and allicin against larva of *Fasciola* in infected snail *Lymnaea acuminata* were tested. Mortality of larvae were observed at 2 h, 4 h, 6 h, and 8 h, of treatment. In *in vivo* treatment, azadirachtin caused highest mortality in redia and cercaria larva (8 h, LC_50_ 0.11, and 0.05 mg/L) whereas in *in vitro* condition allicin was highly toxic against redia and cercaria (8 h, LC_50_ 0.01, and 0.009 mg/L). Toxicity of citral was lowest against redia and cercaria larva.

## 1. Introduction

Fascioliasis is a worldwide zoonotic disease caused by *Fasciola hepatica *and *Fasciola gigantica* (family fasciolidae) [1]. *F. hepatica* has worldwide distribution but predominates in temperate zones while *F. gigantica* is found primarily in tropical regions [2–4]. The definite host is very broad and includes many herbivorous mammals including humans. Human fascioliasis has been reported in 51 different countries from five continents [4]. Fascioliasis is now recognized as an emerging human disease. World health organization has estimated that 2.4 million people are infected with *Fasciola *and a further 180 million are at risk of infection [1]. Singh and Agarwal [5] reported that 94% of buffaloes slaughtered in local slaughtered house in Gorakhpur district are infected with *F. gigantica.* In northern India *Lymnaea acuminata* is the intermediate hosts of the *Fasciola* species [2]. Although control of snail population below a threshold level is one of the important methods for effective control of fascioliasis [6–9], yet snails are one of the important components in the aquatic ecosystem. Release of molluscicides in aquatic system for snail control also affects the other nontarget organism. The *Fasciola *larval stage sporocyst, redia, and cercaria in the snail body are in division phase of *F. gigantica*. If these larvae will be destroyed by plant molluscicides at sublethal concentration in the snail body, the rate of infection can be reduced without killing the snail. Different plants-derived molluscicides and their active component such as citral, ferulic acid, umbelliferone, azadirachtin, and allicin [8, 10–12] were tested against *Fasciola *larva in *in vivo* and *in vitro *condition. There are new approaches to reduce incidence of the fascioliasis without killing the intermediate host snail.

## 2. Material and Methods

### 2.1. Animals

Adult *Lymnaea acuminata* (2.6 ± 0.20 cm in length) were collected locally, and cercaria shedding infected and uninfected snails were separated in two groups. The snails were allowed to acclimatize for 24 hours in laboratory condition. Each infected snail was dissected in a glass petri dish containing 10 mL of dechlorinated water at 22°C–24°C. The pH of the water was 7.1–7.3, and dissolved oxygen, free carbon dioxidez and bicarbonate alkalinity were 6.5–7.2 mg/L, 5.2– 6.3 mg/L, and 102.0–105.0 mg/L, respectively.

After dissection redia and cercaria were separated in a different petri dish containing 10 mL of dechlorinated water. These larvae were kept in dechlorinated tap water where they survive up to 48 h in laboratory condition. 

### 2.2. Plants

*Zingiber officinal*e rhizome, *Allium sativum* bulbs, and *Ferula asafoetida* latex were purchased from local market of Gorakhpur. *Azadirachta indica* oil was supplied by Indian herbs, Saharanpur, India.

### 2.3. Chemicals

Citral, ferulic acid, umbelliferone, azadirachtin, and diallyl disulfide were purchased from Sigma chemical Co., USA allicin was prepared by the method of V.K. Singh and D.K. Singh [11]. 

### 2.4. Toxicity Determination

#### 2.4.1. In Vivo

*In vivo* toxicity of active components at different concentration were determined against larvae of *Fasciola* in infected *Lymnaea acuminata* (Table 1). After 2 h, 4 h, 6 h, and 8 h of treatment, infected snails were dissected. Then live and dead redia and cercaria were counted. Mortality of radia/cercaria was established by immediate arrest of locomotion/movement. It was continuously monitored up to 48 h in all treatments to ensure death. Percent mortality of larvae at each concentration for 2 h, 4 h, 6 h, and 8 h was used for determination of LC_50_. 

#### 2.4.2. In Vitro

*In vitro* toxicity of plant products were performed in the petri dish. Ten redia and cercaria larva of *Fasciola* were separated in different petri dishes containing 10 mL dechlorinated tap water. Treatment of different plant products and their active components was made directly in the petri dish, containing 10 redia/cercaria. Mortality of redia and cercaria were observed after 2 h, 4 h, 6 h, and 8 h of treatment. Counting of larvae was performed with help of a microscope. 

 Lethal value (LC_50_), low and upper confidence limits (LCL and UCL), slope values, *t*-ratio, *g*-value, and heterogeneity factor were calculated with the help of POLO computer programmed by Robertson et al. [13]. One-way ANOVA and product moment correlation coefficient were applied by the method of Sokal and Rohlf [14]. 

## 3. Results

*In vivo* and *in vitro* larvicidal activity of *Z. officinale, F. asafoetida, A. indica, A. sativum, *and their active molluscicides components against the redia and cercaria larva of *F. gigantica *is time and concentration dependent (Tables 2–5). In *in vivo* and *in vitro* treatment azadirachtin and allicin caused highest toxicity against redia and cercaria larva. 8 h LC_50_ of azadirachtin against redia/cercaria larva in *in vivo *treatment was 0.11 mg/L/0.09 mg/L, respectively (Tables 2 and 3). In *in vitro *treatment 8 h LC_50_ of allicin against redia and cercaria was 0.01 and 0.009 mg/L, respectively (Tables 4 and 5). Toxicity of citral against both the larval stage was lowest (Tables 2–5). Significant (*P* < 0.05) negative regression was observed between exposure period and LC_50_ of different plant products. 

The slope values were steep, and separate estimation of LC based on each six replicatewas found with in the 95% confidence limit of LC_50_. The *t*-ratio was greater than 1.96 and the heterogeneity less than 1.0. The *g*-value was less than 0.5 at all probability levels (90, 95, and 99 resp.,); Tables 2, 3, 4, and 5).

## 4. Discussion

Results of the present study clearly indicate that the *Zingiber officinale* (citral), *Ferula asafoetida* (ferulic acid, umbelliferone), *Azadirachta indica* oil (azadirachtin), and *Allium sativum* (allicin) have sufficient larvicidal activity against different larva of *Fasciola gigantica *in *in vivo* and* in vitro *treatments. The alcoholic extract of *A. sativum *bulb has also shown moderate* in vitro *anthelmintic activity against human *Ascaris lumbricoides* [15].* A. sativum* has been reported to be effective in dysentery and also acts as vermifuge [16, 17]. Oil of *A. sativum *has also been reported to possess anthelmintic activity [18–20] and discards all injurious parasites in the intestine [16]*. In vitro *toxicity of allicin against redia (8 h, LC_50_, 0.01 mg/L) and cercaria (8 h, LC_50_, 0.009 mg/L) is highest in *in vitro *condition. 

The steep slope value indicates that a small increase in the concentration of different larvicide caused higher larval mortality. A *t*-ratio value greater than 1.96 indicates that the regression is significant. Heterogeneity factor value less than 1.0 denote that in the replicate test of random sample the concentration response limits and, thus, the model fits the data adequately. The index of significance of the potency estimation *g* indicates that the value of mean is within the limit at all probability level (90, 95, and 99, resp.) since it is less than 0.5. 

*Zingiber officinale* is a perennial plant and is considered to be the universal medicine in Ayurveda. Significant anthelmintic activity of ethanolic extracts of rhizomes of *Zingiber officinale* against *Ascaris lumbricoides *has been reported [15, 21]. Goto et al., [22] observed the *in vitro* lethal effect of *Zingiber officinale *on *Anisakis* larvae. The antifilarial effect of *Z. officinale* against *Dirofilaria immitis *has been reported by Datta and Sukul [23]. Adewunmi et al. [24] and Singh et al. [12] have reported the molluscicidal activity of *Z. officinale*.

Ferulic acid and umbelliferone are (*Ferula asafoetida*) potent molluscicides against *L. acuminata* [7, 8]. Although the antioxidant, anticarcinogenic, antispasmodic, antihelminthic activity of *F. asafoetida *extract and ferulic acid were reported by various workers [25–28], yet there was no report in *in vivo *and* in vitro* larvicidal activity of these active components against (redia and cercaria larva). Azadirachtin, an active component of* Azadirachta indica *inhibits the motility of *Haemonchus contortus *larva [29], Singh et al. [6] observed that *A. Indica *have sufficient molluscicides activity against *L. acuminata.* In *in vivo *treatment of azadirachtin caused highest toxicity against redia (8 h, LC_50_, 0.11 mg/L) and cercaria (8 h, LC_50_, 0.05 mg/L). Present study clearly demonstrates that different larval stages in snail body as well as outside of snail body can be killed without killing the snails. Earlier it has been reported that citral (24 h, LC_50_—68.95 mg/L), ferulic acid (24 h, LC_50_—2.21 mg/L), umbelliferone (24 h, LC_50_—3.43 mg/L), azadirachtin (24 h, LC_50_—0.35 mg/L), and allicin (24 h, LC_50_—6.34 mg/L) are active molluscicides against *L. acuminata* [7, 10–12] 8 h LC_50_ of these plants against redia and cercaria larva is many time low that is used to kill in intermediate host *L. acuminata*. The concentrations that were used to kill redia and cercaria are not toxic to snails, even in 24 h exposure period. Consequently, phytotherapy of snails by these plants and their active component kills the redia and cercaria of *F. gigantica*, without killing the host snail. Snails are a crucial component of an aquatic ecosystem. *In vivo *and *in vitro* killing of redia and cercaria of *F. gigantica* is beneficial as it kills the target larva of *F. gigantica.* Generally active components, that is, citral, ferulic acid, umbelliferone, azadirachtin, and allicin, inhibit activity of acetylcholinesterase, acid/alkaline phosphates, and ATPase in the nervous tissue of *L. acuminata* [8, 11, 30]. In cercaria larva acetylcholinesterase (AChE) in nervous functioning and cytochrome oxidase system in electron transport is well developed for efficient release of energy in active cercaria [31–33]. To elucidate the mechanism of the larvicidal activity of active components against larval stages of *F. gigantica*, their effect on AChE and cytochrome oxidase in larva is required for further investigation.

## 5. Conclusion

It can be concluded from the present study that sublethal treatment of active molluscicidal components citral, ferulic acid, umbelliferone, azadirachtin, and allicin, kill the redia and cercaria larva of *F. gigantica* inside the body of snail *L. acuminata. *Phytotherapy of infected snails by these active components is one of the new method to control the fascioliasis without killing the vector snail, an important components of the aquatic ecosystem.

## Figures and Tables

**Table 1:** Concentration of different active components of plants products used in toxicity trial against *Fasciola gigantica* larva (redia and cercaria).

Larvicides	*In vivo *concentrations mg/L	*In vitro *concentrations mg/L
*Z. officinale* extract	—	7, 10, 20, 30
*F. asafoetida* extract	—	1, 2, 3, 5
Neem oil	—	0.5, 1, 2, 3
*A. sativum* extract	—	0.1, 0.2, 0.3, 0.5
Citral	10, 20, 30, 50	5, 10, 20, 30
Ferulic acid	0.7, 1, 2, 3	0.5, 0.7, 1, 2
Umbelliferone	1, 2, 3, 5	0.3, 0.5, 1, 2
Azadirachtin	0.1, 0.2, 0.3, 0.5	0.07, 0.1, 0.2, 0.3
Allicin	0.7, 1, 3, 5	0.01, 0.02, 0.03, 0.05

**Table 2:** *In vivo* toxicity of different components of plant products against the redia larva of *Fasciola gigantica. *

Exposure	Larvicidal (mg /L)	LC_50_	LCL	UCL	Slope value	*t*-ratio	*g*-value	Heterogeneity
2 h	Citral	59.61	42.39	131.00	1.55 ± 0.36	4.24	0.21	0.12
	Ferulic acid	3.06	2.41	4.67	2.10 ± 0.38	5.43	0.13	0.16
	Umbelliferone	3.70	2.87	5.72	1.56 ± 0.34	4.54	0.18	0.11
	Azadirachtin	1.00	0.52	25.19	0.95 ± 0.35	2.82	0.48	0.14
	Allicin	5.89	3.55	21.65	9.99 ± 0.35	9.80	0.26	0.15

4 h	Citral	39.28	29.88	65.66	1.46 ± 0.34	4.31	0.20	0.18
	Ferulic acid	2.23	1.72	3.53	1.51 ± 0.34	4.41	0.19	0.11
	Umbelliferone	2.48	1.83	3.46	1.34 ± 0.32	4.07	0.23	0.13
	Azadirachtin	0.35	0.25	0.72	1.13 ± 0.32	3.44	0.32	0.10
	Allicin	2.14	1.46	3.42	1.01 ± 0.24	4.22	0.21	0.12

6 h	Citral	26.29	19.48	37.97	1.31 ± 0.32	3.99	0.24	0.23
	Ferulic acid	1.43	1.01	2.02	1.25 ± 0.33	3.74	0.27	0.13
	Umbelliferone	1.52	0.98	1.97	1.52 ± 0.33	4.53	0.18	0.16
	Azadirachtin	0.21	0.13	0.32	1.05 ± 0.32	3.27	0.35	0.11
	Allicin	0.94	0.49	1.35	1.09 ± 0.24	4.43	0.19	0.18

8 h	Citral	13.21	5.35	18.85	1.10 ± 0.32	3.37	0.33	0.20
	Ferulic acid	0.76	0.49	0.98	1.79 ± 0.36	4.94	0.15	0.14
	Umbelliferone	1.11	0.63	1.47	1.64 ± 0.35	4.70	0.17	0.17
	Azadirachtin	0.11	0.06	0.15	1.57 ± 0.34	4.57	0.18	0.25
	Allicin	0.66	0.40	0.90	1.61 ± 0.28	5.67	0.11	0.35

LCL: lower confidence limits, UCL: upper confidence limits. Six batches of 15 snails were exposed to different concentrations of the above molluscicidal treatments. Mortality of redia was recorded every 2 h. Concentrations given are the final concentration (W/V) in the glass aquarium water. Significant negative regression (*P* < 0.05) was observed between exposure time and LC_50_ of treatments. TS: testing significant of the regression coefficient. Citral: 12.06^+^, ferulic acid: 29.31^+^, umbelliferone: 7.76^++^, azadirachtin: 15.58^++^, allicin: 19.19^++^: linear regression between *x* and *y*, ^++^: nonlinear regression.

**Table 3:** *In vivo* toxicity of different active components of plants products against the cercaria larva of *Fasciola gigantica. *

Exposure	Larvicidal (mg/L)	LC_50_	LCL	UCL	Slope value	*t*-ratio	*g*-value	Heterogeneity
2 h	Citral	49.03	34.81	112.16	1.30 ± 0.34	3.80	0.26	0.14
	Ferulic acid	2.53	0.13	4.19	1.57 ± 0.35	4.50	0.19	0.20
	Umbelliferone	3.76	2.71	7.62	1.17 ± 0.33	3.56	0.30	0.17
	Azadirachtin	0.43	0.31	0.90	1.29 ± 0.33	3.81	0.26	0.13
	Allicin	2.80	1.75	7.00	0.79 ± 0.23	3.32	0.34	0.13

4 h	Citral	24.33	18.14	33.16	1.40 ± 0.33	4.24	0.21	0.13
	Ferulic acid	1.65	1.26	2.31	1.43 ± 0.33	4.23	0.21	0.17
	Umbelliferone	1.64	0.87	2.28	1.14 ± 0.32	3.50	0.31	0.14
	Azadirachtin	0.23	0.16	0.32	1.22 ± 0.32	3.75	0.27	0.11
	Allicin	1.06	0.50	1.60	0.92 ± 0.24	3.82	0.26	0.16

6 h	Citral	14.63	9.92	18.53	1.70 ± 0.34	4.46	0.15	0.12
	Ferulic acid	0.98	0.65	1.26	1.53 ± 0.34	4.46	0.19	0.18
	Umbelliferone	1.09	0.45	1.53	1.34 ± 0.33	3.96	0.24	0.09
	Azadirachtin	0.14	0.07	0.19	1.26 ± 0.33	3.82	0.26	0.12
	Allicin	0.42	0.10	0.74	1.02 ± 0.26	3.87	0.25	0.29

8 h	Citral	8.65	3.24	12.73	1.37 ± 0.34	3.94	024	0.19
	Ferulic acid	0.59	0.27	0.82	1.54 ± 0.36	4.21	0.21	0.21
	Umbelliferone	0.92	0.61	1.16	1.34 ± 0.33	5.78	0.11	0.30
	Azadirachtin	0.09	0.05	0.12	1.94 ± 0.37	5.13	0.14	0.39
	Allicin	0.38	0.16	0.58	1.65 ± 0.34	4.84	0.16	0.44

LCL: lower confidence limits, UCL: upper confidence limits. Citral, ferulic acid, umbelliferone, neem oil, azadirachtin, and allicin. Six batches of 15 snails were exposed to different concentration of the above molluscicidal treatments. Mortality of cercaria was recorded every 2 h. Concentrations given are the final concentration (W/V) in the glass aquarium water. Significant negative regression (*P* < 0.05) was observed between exposure time and LC_50_ of treatments. TS: testing significant of the regression coefficient. Citral: 11.02^++^, ferulic acid: 8.35^+^, umbelliferone: 1.56^++^, azadirachtin: 11.82^++^, allicin: 8.52^++^: linear regression between *x* and *y*, ^++^: nonlinear regression.

**Table 4:** *In vitro* toxicity of different plant products and their active components of plants against the redia larva of *Fasciola gigantica. *

Exposure	Larvicidal (mg/mL)	LC_50_	LCL	UCL	Slope-value	*t*-ratio	*g*-value	Heterogeneity
2 h	Z. O. Ext*	35.39	25.96	68.41	1.73 ± 0.37	4.58	0.18	0.29
	Citral	43.21	30.28	91.53	1.71 ± 0.36	4.75	0.17	0.27
	F. A. Ext*	5.58	3.96	12.53	1.45 ± 0.35	4.07	0.23	0.14
	Ferulic acid	1.96	1.41	4.51	1.47 ± 0.38	3.82	0.26	0.24
	Umbelliferone	4.53	2.70	22.59	1.04 ± 0.30	3.45	0.32	0.19
	Neem oil	6.81	3.69	48.35	1.16 ± 0.33	3.52	0.31	0.14
	Azadirachtin	0.38	0.24	1.42	1.20 ± 0.35	3.42	0.32	0.17
	A. S. Ext*	0.81	0.52	2.99	1.42 ± 0.37	3.76	0.27	0.21
	Allicin	0.05	0.04	0.15	1.31 ± 0.34	3.76	0.27	0.13

4 h	Z. O. Ext	24.46	18.98	38.64	1.63 ± 0.35	4.66	0.17	0.13
	Citral	19.59	14.72	30.48	1.32 ± 0.29	4.56	0.18	0.19
	F. A. Ext	3.98	2.79	9.71	1.09 ± 0.33	3.31	0.35	0.12
	Ferulic acid	1.16	0.88	1.89	1.32 ± 0.37	3.56	0.30	0.16
	Umbelliferone	1.85	1.35	3.00	1.20 ± 0.28	4.66	0.17	0.13
	Neem oil	2.62	1.92	4.76	1.35 ± 0.29	4.52	0.18	0.16
	Azadirachtin	0.14	0.10	0.18	1.39 ± 0.33	4.14	0.22	0.15
	A. S. Ext	0.57	0.37	2.22	1.11 ± 0.33	3.29	0.35	0.13
	Allicin	0.03	0.02	0.07	1.20 ± 0.33	3.63	0.29	0.15

6 h	Z. O. Ext	13.88	10.37	18.16	1.49 ± 0.33	4.40	0.19	0.18
	Citral	0.97	5.38	12.29	1.96 ± 0.28	4.22	0.21	0.13
	F. A. Ext	1.45	0.83	1.93	1.34 ± 0.33	4.06	0.23	0.23
	Ferulic acid	0.69	0.50	0.85	1.81 ± 0.39	4.64	0.17	0.13
	Umbelliferone	0.77	0.49	1.03	1.40 ± 0.28	4.86	0.16	0.14
	Neem oil	1.24	0.87	1.71	1.25 ± 0.28	4.41	0.19	0.15
	Azadirachtin	0.10	0.07	0.12	1.92 ± 0.35	5.45	0.12	0.13
	A. S. Ext	0.21	0.13	0.31	1.11 ± 0.32	5.94	0.32	0.11
	Allicin	0.01	0.01	0.02	1.59 ± 0.33	4.78	0.16	0.14

8 h	Z. O. Ext	8.55	6.30	10.43	2.14 ± 0.37	5.79	0.11	0.18
	Citral	4.14	0.02	1.22	1.27 ± 0.30	4,21	0.21	0.22
	F. A. Ext	0.85	0.53	1.09	2.46 ± 0.44	5.53	0.12	0.41
	Ferulic acid	0.45	0.29	0.57	2.30 ± 0.04	4.99	0.15	0.41
	Umbelliferone	0.63	0.41	0.82	1.75 ± 0.30	5.68	0.11	0.19
	Neem oil	0.71	0.47	0.92	1.61 ± 0.29	5.40	0.13	0.40
	Azadirachtin	0.07	0.05	0.09	1.92 ± 0.37	5.31	0.13	0.18
	A. S. Ext	0.11	0.03	0.17	1.04 ± 0.32	3.19	0.37	0.11
	Allicin	0.01	0.01	0.02	1.66 ± 0.33	4.94	0.15	0.17

Z. O. Ext: *Zingiber officinale*, F. A. Ext: *Ferula asafoetida* extract, A. S. Ext: *Allium sativum* extract, LCL: lower confidence limits, UCL: upper confidence limits. Six batches of 10 redia larva were exposed to different concentration of the above molluscicides treatments. Mortality was recorded every 2 h. Concentrations given are the final concentration (W/V) in the glass aquarium water. Significant negative regression (*P* < 0.05) was observed between exposure time and LC_50_ of treatments. TS: testing significant of the regression coefficient. Z. O. Ext: 9.58^+^, citral: 7.18^++^, ferulic asafetida: 6.51^+^, ferulic acid 8.68^++^, umbelliferone: 10.52^++^, neem oil: 16.46^++^, azadirachtin: 13.34^++^, *allium sativum*: 1.57^+^, allicin: 4.43^++^: linear regression between *x* and* y*, ^++^: nonlinear regression.

**Table 5:** *In vitro* toxicity of different plant products and their active components against the cercaria larva of *Fasciola gigantica. *

Exposure	Larvicidal (mg/mL)	LC_50_	LCL	UCL	Slope value	*t*-ratio	*g*-value	Heterogeneity
2 h	Z. O. Ext	33.77	22.98	99.35	1.24 ± 0.34	3.56	0.30	0.11
	Citral	39.99	28.86	76.90	1.11 ± 0.30	4.95	0.15	0.27
	F. A. Ext	4.51	3.13	12.15	1.70 ± 0.32	3.37	0.81	0.55
	Ferulic acid	1.72	1.21	5.18	1.18 ± 0.37	3.18	0.37	0.13
	Umbelliferone	1.49	1.06	2.83	1.23 ± 0.27	4.45	0.19	0.18
	Neem oil	6.81	3.13	12.15	1.09 ± 0.28	3.91	0.21	0.15
	Azadirachtin	0.38	0.26	0.87	1.57 ± 0.37	4.22	0.29	0.19
	A. S. Ext	0.39	0.28	0.84	1.19 ± 0.33	3.59	0.29	0.12
	Allicin	0.03	0.02	0.07	1.21 ± 0.33	3.64	0.28	0.13

4 h	Z. O. Ext	14.91	10.67	21.43	1.25 ± 0.33	3.74	0.27	0.17
	Citral	19.81	14.72	31.76	1.27 ± 0.28	4.42	0.19	0.14
	F. A. Ext	2.44	1.72	3.57	120 ± 0.32	3.69	0.28	0.10
	Ferulic acid	0.95	0.62	1.58	1.07 ± 0.36	2.91	0.45	0.16
	Umbelliferone	1.03	0.70	1.97	1.97 ± 0.26	3.62	0.29	0.14
	Neem oil	1.41	0.91	4.11	1.89 ± 0.26	3.34	0.34	0.12
	Azadirachtin	0.27	0.20	0.51	1.51 ± 0.35	4.30	0.20	0.15
	A. S. Ext	0.22	0.17	0.29	1.48 ± 0.33	4.47	0.19	0.18
	Allicin	0.02	0.01	0.03	1.21 ± 0.32	3.71	0.27	0.14

6 h	Z. O. Ext	9.49	5.94	12.38	1.45 ± 0.34	4.22	0.21	0.15
	Citral	8.73	5.60	11.62	1.33 ± 0.28	4.83	0.17	0.21
	F. A. Ext	1.03	0.16	1.63	1.78 ± 0.32	2.42	0.65	0.11
	Ferulic acid	0.51	0.23	0.70	1.34 ± 0.38	3.47	0.31	0.12
	Umbelliferone	0.46	0.32	0.59	1.61 ± 0.28	5.41	0.12	0.16
	Neem oil	0.62	0.40	0.89	1.10 ± 0.27	4.79	0.23	0.14
	Azadirachtin	1.56	0.12	0.21	1.51 ± 0.33	4.46	0.19	0.12
	A. S. Ext	0.14	0.08	0.18	1.46 ± 0.33	4.41	0.19	0.57
	Allicin	0.07	0.04	0.01	1.17 ± 0.33	3.53	0.30	0.34

8 h	Z. O. Ext	7.73	5.03	9.84	1.83 ± 0.36	5.02	0.15	0.22
	Citral	6.08	4.23	7.69	2.02 ± 0.32	6.21	0.10	0.38
	F. A. Ext	0.99	0.68	1.24	2.49 ± 0.47	5.98	0.10	0.30
	Ferulic acid	0.44	0.29	0.55	2.44 ± 0.48	4.99	0.13	0.25
	Umbelliferone	0.27	0.13	0.39	1.39 ± 0.30	4.63	0.17	0.20
	Neem oil	0.31	0.17	0.44	1.40 ± 0.29	4.75	0.17	0.16
	Azadirachtin	0.08	0.05	0.11	1.50 ± 0.34	4.34	0.20	0.21
	A. S. Ext	0.12	0.09	0.15	2.22 ± 0.36	6.01	0.10	0.26
	Allicin	0.009	0.005	0.01	1.75 ± 0.36	4.74	0.17	0.25

Z. O. Ext: *Zingiber officinale*, F. A. Ext: *Ferula asafoetida extract*, A. S. Ext: *Allium sativum* extract, LCL: lower confidence limits, UCL: upper confidence limits. Six batches of 10 cercaria larva were exposed to different concentration of the above molluscicides treatments. Mortality of cercaria was recorded every 2 h. Concentration given are the final concentration (W/V) in the glass aquarium water. Significant negative regression (*P* < 0.05) was observed between exposure time and LC_50_ of treatments. TS: testing significant of the regression coefficient. Z. O. Ext: 17.91^++^, citral: 9.52^++^, ferulic asafetida: 6.04^++^, ferulic acid: 9.95^++^, umbelliferone: 7.69^+^, neem oil: 57.95^++^, azadirachtin: 13.39^+^, *allium sativum*: +6.29^++^, allicin: 5.81^++^: linear regression between x and y, ^++^: nonlinear regression.

## References

[1] World Health Organization (2006). *Report of the WHO Informal Meeting on Use of Triclabendazole in Fascioliasis
Control*.

[2] Agarwal RA, Singh DK (1988). Harmful gastropods and their control. *Acta Hydrochimica et Hydrobiologica*.

[3] Mas-Coma S, Bargues MD, Valero MA (2005). Fascioliasis and other plant-borne trematode zoonoses. *International Journal for Parasitology*.

[4] Mas-Coma MS, Esteban JG, Bargues MD (1999). Epidemiology of human fascioliasis: a review and proposed new classification. *Bulletin of the World Health Organization*.

[5] Singh O, Agarwal RA (1981). Toxicity of certain pesticides to two economic species of snail in northern India. *Journal of Economic Entomology*.

[6] Singh A, Singh DK, Misra TN, Agarwal RA (1996). Molluscicides of plant origin. *Biological Agriculture and Horticulture*.

[7] Kumar P, Singh DK (2006). Molluscicidal activity of *Ferula asafoetida*, *Syzygium aromaticum* and *Carum carvi* and their active components against the snail *Lymnaea acuminata*. *Chemosphere*.

[8] Kumar P, Singh VK, Singh DK (2009). Kinetics of enzyme inhibition by active molluscicidal agents ferulic acid, umbelliferone, eugenol and limonene in the nervous tissue of snail *Lymnaea acuminata*. *Phytotherapy Research*.

[9] Jaiswal P, Singh DK (2009). Molluscicidal activity of nutmeg and mace (*Myristica fragrans* houtt.) against the vector snail *Lymnaea acuminata*. *Journal of Herbs, Spices and Medicinal Plants*.

[10] Singh K, Singh A, Singh DK (1995). Molluscicidal activity of different combinations of the plant products used in the molluscicide Pestoban. *Biological Agriculture and Horticulture*.

[11] Singh VK, Singh DK (1995). Characterization of allicin as a molluscicidal agent in *Allium sativum* (Garlic). *Biological Agriculture and Horticulture*.

[12] Singh S, Singh VK, Singh DK (1997). Molluscicidal activity of some common spice plants. *Biological Agriculture and Horticulture*.

[13] Robertson JL, Russell RM, Preciter HK, Savin NE (2007). *Bioassay with Arthropods Data*.

[14] Sokal RR, Rohlf FJ (1996). *Introduction of Biostatistics*.

[15] Kaleysa Raj R (1975). Screening of indigenous plants for anthelmintic action against human *Ascaris lumbricoides*: part II. *Indian Journal of Physiology and Pharmacology*.

[16] Nadkarni KM (1976). *Indian Materia Medica. Vol I and II Popular Prakashan*.

[17] Schavenberg P, Paris F (1977). *Guide to Medicinal Plants*.

[18] Steenis-Kruseman MJV (1953). Select Indonesian medicinal plants organize. *Science Research Indonesia Bulltin*.

[19] Hoppe HA (1975). *Drogenkunde, Vol. I .Angiosperms*.

[20] Kirtikar KR, Basu BD (1981). *Indian Medicinal Plants. Part II*.

[21] Kaleysa Raj R (1974). Screening of some indigenous plants for anthelmintic action against human *Ascaris lumbricoides*. *Indian Journal of Physiology and Pharmacology*.

[22] Goto C, Kasuya S, Koga K, Ohtoma H, Kagei N (1990). Lethal efficacy of extract from *Zingiber officinale* (traditional Chinese medicine) or [6]-shogaol and [6]-gingerol in *Anisakis* larvae *in vitro*. *Parasitology Research*.

[23] Datta A, Sukul NC (1987). Antifilarial effect of *Zingiber officinale* on *Dirofilaria immitis*. *Journal of Helminthology*.

[24] Adewunmi CO, Oguntimein BO, Furu P (1990). Molluscicidal and antischistosomal activities of *Zingiber officinale*. *Planta Medica*.

[25] Eigner D, Scholz D (1999). *Ferula asa-foetida* and *Curcuma longa* in traditional medical treatment and diet in Nepal. *Journal of Ethnopharmacology*.

[26] Saleem M, Alam A, Sultana S (2001). *Asafoetida* inhibits early events of carcinogenesis: a chemopreventive study. *Life Sciences*.

[27] Furukawa H, Zenno S, Iwasawa Y, Morita H, Yoshida T, Nagasawa T (2003). Ferulic acid production from clove oil by pseudomonas fluorescens E118. *Journal of Bioscience and Bioengineering*.

[28] Fatehi M, Farifteh F, Hassanabad ZF (2004). Antispasmodic and hypotensive effects of Ferula *Asafoetida* gum extract. *Journal of Ethnopharmacology*.

[29] Assis LM, Bevilequa CML, Morais SM, Vieira LS, Costa CTC, Souza JAL (2003). Ovicidal and larvicidal activity *in vitro* of *Spieled anthelia* Linn. Extract on *Haemonchus contorlus*. *Veterinary Parasitology*.

[30] Singh VK, Singh S, Singh S, Singh DK (1999). Effect of active molluscicidal component of spices on different enzyme activities and biogenic amine levels in the nervous tissue of *Lymnaea acuminata*. *Phytotherapy Research*.

[31] Humiczewska M (1975). Oxidative enzymes in the development of *Fasciola hepatica* L. *Folia Histochemica et Cytochemica*.

[32] Tielens AGM, Fried B, Graczyk TK (1997). Biochemistry of trematodes. *Advances in Trematode Biology*.

[33] Schmidt GD, Roberts LS (2000). *Trematoda: From Function, and Classification of Digeneans Foundation of Parasitology*.

